# The Eight-Hour Law

**Published:** 1872-02

**Authors:** 


### THE EIGHT-HOUR LAW

Means that a man who wants to build a house, or have any other work done, must pay the laborer as much for working eight hours as if he worked all day, "from sun to sun." That is, the laborer demands more for his work than it is worth. And as one wrong sooner or later makes another wrong necessary to sustain it, those who think differently, and are willing to work all day for a day's wages, are told that if they do so they will have their heads broken, or their bodies beat to a jelly.

Here, then, comes out in its strongest colors the despotism of labor, its unreasoning tyranny; it compels the moneyed man to pay more for his work than it is worth, and forces a fellow laborer to remain idle, when he wants to work, and when, unless he works, his wife and children will have nothing to eat to-morrow, and they must steal or starve. This is only another phase of

COMMUNISM, taking a less offensive name, calling themselves "Internationals," that is, the banding together of the "Eight-Hour" men of all nations, for the purpose of carrying out their views all over the world.

The "Eight-Hour" men, the Communists, the Internationals, are essentially one and the same; as a class, they are the common enemies of law and liberty, and are at heart despots, thieves, and murderers. We do not mean to say that the Eight-Hour men in the United States are bad men now; they do not intend to be bad men; but their principles, carried out legitimately, end in the worst of human crimes, as the most beautiful city in the world has verified within the year.

An Eight-Hour law makes me pay for a job of work more than I think it is worth. This is despotism in its very worst form. If I refuse, and go in search of a man who will work for a more moderate compensation, and who is willing to do so, he is threatened with death if he undertakes it.

But suppose I yield, and pay for eight hours' work as much as twelve hours' work is worth, my house costs me one third more than it is worth; and I am compelled to charge three thousand dollars a year for its rent, when otherwise I should have charged but two thousand; and three hundred for a cottage, instead of two hundred; or a hundred and fifty for a set of rooms, instead of a hundred; and so with everything else; for the cost of everything is regulated by the cost of dwelling, factory, store, and shop.

But if a man gets one third more for his work, and has to pay one third more for what he wants, house-rent, food, and clothing, he is no better off than before; he is rather worse off; he is going behindhand every day, because his family wears out as much clothing, and eats as much food, in his working eight hours as in working twelve, while he does not earn as much for eight hours' work as for twelve, that is, he loses one third of his time; one third of his time he is idle ; you may call it rest, recreation, opportunity for mental improvement, or anything else you please, that time yields him no money; if he was working, it would yield him money or money's worth. Any intelligent mechanic or laborer will tell you that the times are harder now than when he received half as much for his work as to-day.

The real argument runs thus: No man can do as much work in a week, by laboring eight hours a day, as if he worked from sunrise to sunset. Yet his family eats as much, and wears out as much clothing; hence he is getting behindhand, financially, every hour. He gets as much money for his eight hours as he used to get for ten or twelve, but house-rent, and food, and clothing, cost more; hence, unless he works more than eight hours, he is living beyond his means. Every mechanic will tell you that it is harder to get along now than it used to be, in consequence of the increased cost of everything.

The higher the wages, the worse it is for the workmen. This is an apparent absurdity; but hard facts point it out in characters which cannot be mistaken. If the mass of workmen are paid as much for half a day's labor as for a whole day, and according to the laws of "strikes" a longer time of labor is not allowed, then there is half a day's idleness, and in its train, vice and crime, as certain as the rising of the sun. One authentic fact is worth any amount of theory.

During the year 1871, a considerable number of Bohemians returned to their native land, who, three years before, felt compelled, from the low wages they were receiving, to emigrate to the United States, induced to do so by the reported high wages which there prevailed. On their arrival they found this to be true; the wages were fabulous, and so were the prices of meat, and bread, and house-rent higher in proportion ; they found that they could live more plentifully on their small wages in Bohemia, than on the four or five times higher wages in America, where it required a week's work to pay for one month's rent.

The best solution on the part of workmen for this phase of the question is, that they be allowed to work more hours, at the same rate.

The plea that the time beyond eight hours a day is needed by the workmen for rest, recreation, mental improvement, and the like, is a falsity, because these extra hours are spent by the multitude in unprofitable, and very often in demoralizing practices.

The newsboy, in his hours of recreation, in his rags and tatters, pitches quoits on a wager, with copper cents, or delights himself at fisticuffs or mumblepeg. The day-laborer, or the mechanic, hangs around the street corners, or visits the grocery, tilts back in a chair, sits on the counter, or stands about the stove whittling soft wood, spitting tobacco-juice, and talks politics by the hour.

A lamentably small number of Eight-Hour men go direct to their homes after the day's work is done, to talk to their children, to read to their families, to help their wives, and to make themselves generally useful about the house. On the contrary, as vice and idleness are known everywhere to go together, the unemployed, in too many cases, spend less and less time at home, become more and more fond of the low talk and the scandal of the grocery ; the fireside of earlier years becomes less and less attractive, and unthrift and drunkenness complete the record of life.

If the relation between the employers and employed becomes so conflicting that the worker, in self-defense, must carry his point, let him be allowed by his fellow-workmen to work any number of hours beyond the eight which suits him, he being paid for the same in proportion.

But the truth is, this " Eight-Hour Law " is a palpable fallacy, and becomes more and more impossible of realization every day, because with every day population increases, and with it, in infallible proportion, competition in business; there are more men to do the same amount of work, more families to be supported from the same piece of ground, and to do that, the ground must be cultivated more diligently, must be worked more effectually, and to do so, requires more time.

Perhaps, after all, the only solution in large cities and working districts, will be found in the principle of

ASSOCIATION, which means that two, or ten, or any other number of men, shall work together for one another, for themselves, and receive the profits of their wo.k, instead of giving their profits to the "Boss," to the one man who carries on the business; then they will find that no business will yield a profit which is not worked at more than eight hours a day. Where is the mechanic's wife, or the farmer's wife, who can get along by working only eight hours a day? The wife has to get the mechanic's breakfast by sunrise in summer, and by daylight in winter. In nine cases out of ten, farmers' wives are up and stirring before the sun, and in too many cases, ten o'clock finds them still out of bed. Is this the Eight-Hour law? Do

William B. Astor,
A. T. Stewart,
Cornelius Vanderbilt,
Moses Taylor,
George Law, or others of the noted men of our time work but eight hours a day? Have their fortunes been built up by eight hours in business occupation? Is there a man of the many whose wealth counts beyond a hundred thousand dollars, who can boast that he has worked but eight hours a day? They will confess, every man of them, to nearer sixteen.

The truth is, civilized society cannot be supported by the workers laboring only eight hours a day. No man, who depends on the labor of his own hands for the support of himself, and wife, and children, can possibly earn enough in eight hours' daily labor, to provide for them necessary food and clothing, even if he had his house free; but house-rent has to be paid — paid to the man who owns it, and who lives on its rent; thus it is that the workers must feed and clothe not only their own families, but the families of those who do not work at all. And if eight hours' daily labor cannot, has not, hitherto, supported their own families, it is not possible to support others besides.

There is only one way of solving the difficult problem; difficult, because the laborer must be protected as well as the capitalist; there is a way in which all can have their just dues. Let the laborer work for so much an hour, and for as many hours in the day as he chooses to work.

In cases where this will not or cannot apply, for example, where it requires more than one person to carry on a business, let as many as desire it, as before stated, combine together, and divide their own profits among themselves, in such proportion as the skill and ability of each is worth; this is what is meant by

COÖPERATION, and is the most just and manly way of solving the much vexed problem, and thus break up all trades unions, internationalism strikes, and "combinations" of labor against capital; the issue of which war will always be, as long as the world stands, in favor of the capitalist; for the fiat has gone forth, coming from the throne of Omnipotence, and it is the fiat of the ages, "In the sweat of thy face shalt thou eat bread."

